# Dose-response meta-analysis of plasma TMAO and stroke: validated linear risk threshold at 3.0 μmol/L

**DOI:** 10.3389/fneur.2026.1749522

**Published:** 2026-01-30

**Authors:** Jiakai Zhang, Tao Yu, Lefang Liu, Ruizhi Luan

**Affiliations:** Multi-Specialty Ward, Maternal and Child Health Care Hospital of Dong'e, Liaocheng, Shandong, China

**Keywords:** cerebrovascular accident, dose response meta-analysis, observational study, stroke, trimethylamine N-oxide

## Abstract

**Background:**

Stroke, especially the ischemic type, remains a leading global cause of death and disability, with modifiable risk factors offering prevention opportunities. Trimethylamine N-oxide (TMAO), a gut-derived metabolite, promotes vascular damage and is linked to stroke risk. Although prior studies have explored dose-response relationships, clinically actionable thresholds remain undefined, limiting translational applications. This study aims to advance the field by quantifying a continuous dose-response relationship and determining a specific risk threshold, which is currently lacking, to inform preventive strategies.

**Methods:**

This PRISMA-compliant meta-analysis included 11 observational studies (*n* = 7,556) and encompassed two components: an overall meta-analysis of 10 studies to compare admission TMAO levels, and a dose-response meta-analysis that was specifically applied to the subset of 4 studies with sufficient data across multiple exposure categories. We pooled standardized mean differences (SMD) for admission TMAO levels and modeled dose-response curves using restricted cubic splines (knots at 2.37/3.45/5.95 μmol/L). Heterogeneity was quantified using the *I*^2^-statistic, sensitivity was assessed using alternative statistical models and dose scaling approaches, and publication bias was evaluated with Egger's test and the trim-and-fill method.

**Results:**

Stroke patients showed significantly higher TMAO vs. controls (SMD = 0.55, 95% CI: 0.35, 0.74; *P* < 0.00001). Linear dose-response relationship: Each 1 μmol/L TMAO increase raised stroke risk by 8.9% (OR = 1.089, 95% CI: 1.023–1.158; *P* = 0.007). Risk threshold: TMAO > 3.0 μmol/L significantly increases the risk (OR > 1) and warrants preventive intervention. Cumulative risk escalated: 0 → 5 μmol/L: 53% risk increase (OR = 1.53); 0 → 20 μmol/L: 448% risk increase (OR = 5.48); robustness confirmed by sensitivity analysis (*I*^2^ = 35.9%; Cochran *Q, P* = 0.154).

**Conclusion:**

TMAO exhibits a linear, dose-dependent association with stroke risk, with ≥ 3.0 μmol/L serving as a critical threshold for clinical intervention.

## 1 Introduction

Stroke is categorized into ischemic and hemorrhagic subtypes, with approximately 87% of cases being ischemic (1). Ischemic stroke (IS) accounts for 80% of all strokes and is one of the most fatal diseases globally (2). The fundamental pathological cause of IS is intravascular thrombosis, leading to cerebral tissue necrosis and focal neuronal deficits (3). Globally, IS caused 188.3 million disability-adjusted life years (DALYs) in 2021, positioning it as the second leading cause of death (4). In the US, IS accounts for approximately 87% of all strokes, representing the fifth leading cause of death and a primary contributor to severe long-term disability (5). In China, incidence surged from 760,000 cases (1990) to 2.77 million cases (2021), the age-standardized rate of DALYs decreased by 0.50%, but the absolute number rose (from 9.92 million to 23.43 million) (6, 7).

Trimethylamine N-oxide (TMAO) is a gut microbiota-derived metabolite. Dietary nutrients rich in phosphatidylcholine and choline—such as red meat, eggs, and to a lesser extent, dairy products like milk—are metabolized by gut microbes into trimethylamine (TMA). TMA enters the portal circulation and is oxidized to TMAO in the liver, primarily by flavin-containing monooxygenases (FMO), especially FMO3 (8, 9). TMAO is eliminated from the body primarily via renal excretion and secondarily through reduction back to TMA by intestinal microbes (10). Studies indicate that renal function is a critical determinant of TMAO accumulation, even mild renal impairment (eGFR < 66 ml/min/1.73 m^2^) can lead to elevated levels of TMAO (11, 12). TMAO exhibits strong associations with cardiovascular and cerebrovascular pathologies. Elevated TMAO levels are associated with reduced expression of tight junction proteins (claudin-5, ZO-1) in experimental models, which may contribute to cerebral small vessel damage (10). Moreover, TMAO promotes atherosclerosis by upregulating scavenger receptors (CD36/SR-A1) on macrophages, thereby accelerating foam cell formation (10, 13, 14). It also positively correlates with pro-inflammatory monocytes (CD14++CD16+; *r* = 0.70), potentially exacerbating thrombosis and vascular inflammation through monocyte activation (15, 16).

Based on the aforementioned pathophysiological mechanisms, several meta-analyses have supported the TMAO-stroke association (17–19), but they yield conflicting conclusions regarding the dose-response shape. Specifically, the meta-analyses by Farhangi et al. (18) identified a non-linear relationship, whereas the meta-analysis by Chen et al. (17) and Hong et al. (19) found a linear one. Building upon prior meta-analyses that identified an association, this study aims to advance the field by a dose-response meta-analysis (DRMA) to determine a specific risk threshold, which is currently lacking, to inform preventive strategies.

## 2 Materials and methods

### 2.1 Search strategy

This DRMA was conducted and reported in accordance with the PRISMA guidelines (20). Literature searches encompassed records from database inception until 1 July 2025, with language restrictions to English or Chinese. The study details have been registered with PROSPERO under number CRD420251151002.

We searched the following databases: PubMed, Web of Science (WOS), Embase, Scopus, Cochrane Library, Sinomed, China National Knowledge Infrastructure (CNKI), WanFang Data, and VIP (China Science and Technology Journal Database). Clinical trial registries: ClinicalTrials.gov and the Chinese Clinical Trial Registry (ChiCTR) to identify ongoing or unpublished trials.

**Figure 1:**
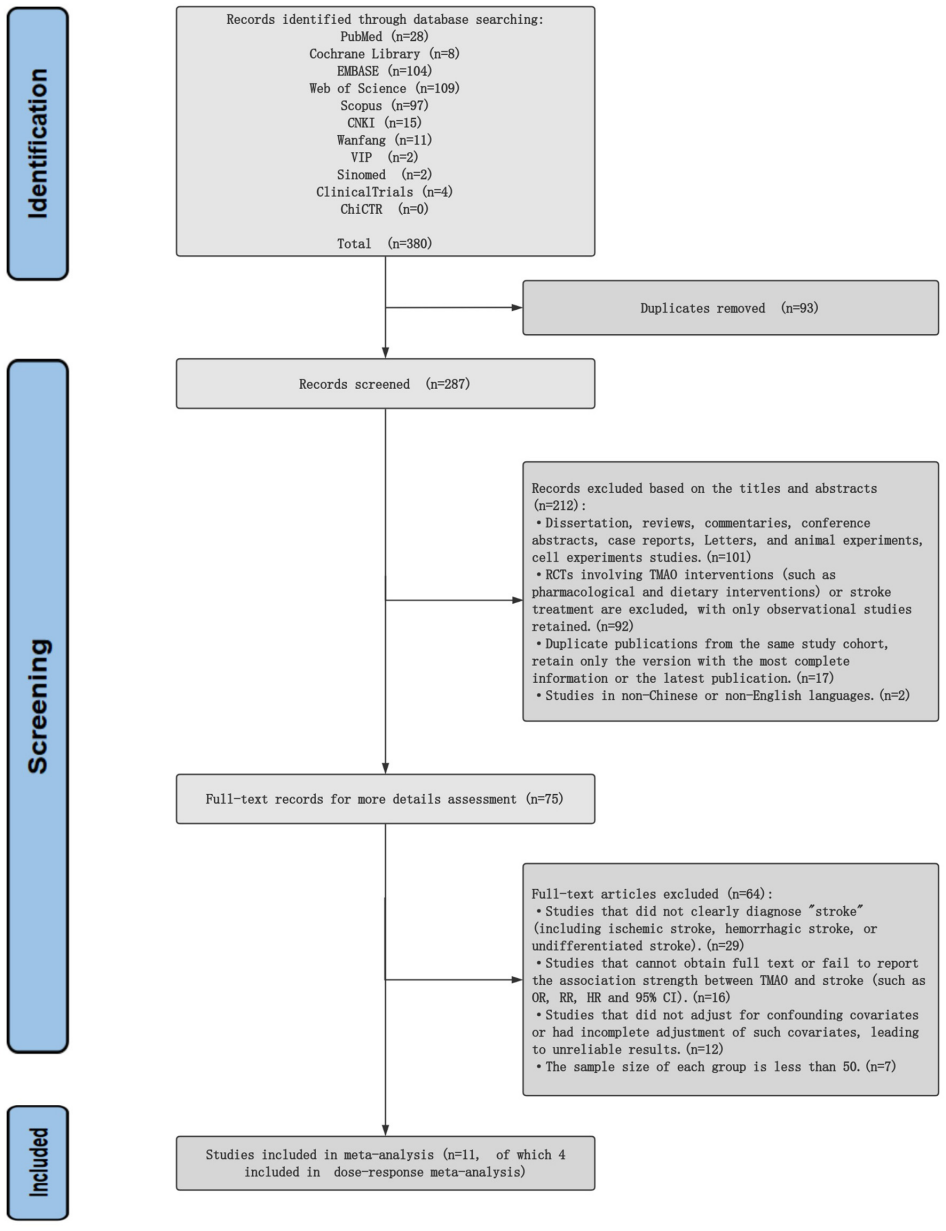
Flow diagram of the literature search and selection process.

Search terms included TMAO, trimethylamine N-oxide, stroke, cerebrovascular accident, CVA, observational study, cohort Study, cross-sectional Study, case-control study, etc. The detailed search strategy is provided in the Supplementary Materials, and a flow diagram is presented in Figure 1.

### 2.2 Inclusion and exclusion criteria

Inclusion criteria: (a) Observational studies (cohort, case-control, or cross-sectional designs); (b) patients with a confirmed stroke diagnosis based on neuroimaging (e.g., MRI or CT), and subsequently classified by etiology using standardized systems such as the TOAST criteria; (c) measurement of blood TMAO levels; (d) provision of accessible TMAO level statistics [e.g., mean/standard deviation (SD) or median/interquartile range (IQR)].

Exclusion criteria: (a) Randomized controlled trials involving TMAO interventions (e.g., drug or dietary interventions) or stroke treatment, as well as dissertations, reviews, commentaries, conference abstracts, case reports, correspondence articles, and animal or cell experimental studies; (b) studies without a clear diagnosis of “stroke” or using unrecognized diagnostic criteria; (c) studies with unreliable results due to unadjusted or incompletely adjusted confounding variables; (d) studies with inaccessible full texts, incomplete data reporting, or duplicate publications; (e) studies with a sample size of < 50 per group.

### 2.3 Study selection and data extraction

Two authors (JZ and LL) independently searched databases and screened titles, abstracts, and full texts to identify eligible articles. Disagreements were resolved through discussion with a third author (RL). Data extraction by two authors (JZ and TY) followed a predefined protocol, capturing information on title, author, country, publication year, group characteristics, outcome measures, results, and conclusions. Disagreements during extraction were resolved by consulting the third author (RL).

### 2.4 Risk of bias assessment

Two reviewers (TY and LL) independently assessed the methodological quality of the included observational studies using the Newcastle–Ottawa Scale (NOS) (21). This tool evaluates studies based on three domains: selection of the study groups (maximum 4 stars), comparability of the groups (maximum 2 stars), and ascertainment of either the outcome or exposure (maximum 3 stars), yielding a total maximum score of 9. A higher score indicates higher study quality, with a score of ≥7 typically indicating a high-quality study. Any disagreements between the reviewers during the assessment were resolved through discussion or adjudication by a third reviewer (RL).

### 2.5 Data analysis

In line with the primary objective of establishing a dose-response relationship, the data analysis proceeded in two stages. First, a standard meta-analysis was performed using Review Manager 5.4 software. Data were presented as means ($\bar{X}$) and SD with 95% CIs. A fixed-effects model (FEM) was used if *I*^2^ < 50% (no significant heterogeneity); otherwise, a random-effects model (REM) was employed. For studies (22–28) reporting medians and IQR, data were converted to $\bar{X}$ and SD using formulas of Wan et al. (29) and Luo et al. (30): $\bar{X}\approx \left(0.7+\frac{0.39}{n}\right)\frac{q_{1}+q_{3}}{2}+\left(0.3-\frac{0.39}{n}\right)m$, $SD\approx \frac{IQR}{1.35}$ (*IQR* = *q*_3_−*q*_1_).

Second, and central to this aim, a DRMA was performed on 4 studies (22, 23, 26, 31) that provided sufficient data on multiple exposure categories, enabling the examination of concentration-response relationships. This analysis was performed using R 4.5.1 with a two-stage random-effects framework. Within-study non-independence across dose groups was addressed using Greenland–Longnecker covariance correction (32). Studies-specific dose-response curves were fitted using weighted least squares. A restricted cubic spline (RCS) model with three knots (positioned at the 10th, 50th, and 90th percentiles of the dose distribution: 2.37, 3.45, 5.95 μmol/L) modeled non-linear relationships. In the between-study stage, maximum likelihood (ML) was used for fixed effects, and restricted maximum likelihood (REML) was used to estimate the random-effects covariance structure. Especially, a key finding of our analysis was the determination of a TMAO risk threshold, defined as the lowest dose at which the lower bound of the 95% CI for the pooled OR first exceeded 1.0. Specifically, the algorithm automatically identified this threshold by scanning dose points in ascending order and selecting the first dose that met this criterion. Heterogeneity was quantified using Cochran's *Q* test (*P* > 0.10 indicating low heterogeneity) and the *I*^2^-statistic (>50% indicating substantial heterogeneity). Traditional leave-one-out sensitivity analysis was precluded, as the study by Nie et al. (23) contributed only two non-referent observations, rendering model fitting unstable upon exclusion of any study. We therefore assessed robustness using alternative strategies, including different statistical methods (fixed-effect and maximum-likelihood models) and dose transformations (log and standardization). Publication bias was assessed visually via funnel plots and quantitatively using Egger's linear regression test. Trim-and-fill analysis was used for bias correction.

## 3 Results

### 3.1 Characteristics of included studies

11 studies (22–28, 31, 33–35) (total *n* = 7,556; 3,878 stroke patients, 3,678 controls) published between 2017 and 2023 were included. 9 studies (22–24, 26–28, 33–35) originated from China, 1 from Germany (25), and 1 from the Spain (31). TMAO was primarily measured via liquid chromatography-tandem mass spectrometry (LC-MS/MS), with 1 study (24) using ultra-high-performance LC-MS/MS (UHPLC-MS/MS). Stroke types, all confirmed by neuroimaging (MRI/CT), included ischemic stroke (24–28, 33–35) and mixed types (22, 23, 31). The TOAST criteria were used for etiological classification of the ischemic strokes. The characteristics of the included studies are presented in Table 1.

**Table 1:** Characteristics of included studies.

**First author, year**	**Country**	**Study design**	**Sample size (cases/controls/cohort)**	**Stroke type**	**TMAO detection method**	**Key outcomes evaluated**	**Adjusted confounders**	**DRMA**
Wu C, 2020	China	Prospective cohort	377 (Ischemic stroke patients) + 50 (Healthy controls)	Acute ischemic stroke	LC-MS/MS	Stroke severity (NIHSS), infarct volume	Age, sex, hypertension, coronary artery disease, atrial fibrillation, smoking, eGFR, etc.	No
Schneider C, 2020	Germany	Prospective case-control	193 (Ischemic stroke patients) + 100 (Controls)	Ischemic stroke	LC-MS/MS	TMAO time course, stroke severity (NIHSS)	Age, sex, hypertension, diabetes, GFR	No
Chen Y-Y, 2022	China	Case-control	291 (LAA ischemic stroke) + 235 (Asymptomatic controls)	Large artery atherosclerotic (LAA) ischemic stroke	LC-MS	Major vascular event recurrence, functional outcome (mRS)	Age, sex, hypertension, diabetes, smoking, creatinine	No
Xu D, 2021	China	Cross-sectional comparative	50 (LAA stroke patients) + 50 (Healthy controls)	Large artery atherothrombotic stroke	LC-MS/MS	Stroke risk, blood lipid-related indices	Age, sex, BMI, smoking, hypertension, eGFR	No
Zhu C, 2019	China	Prospective cohort	256 (Acute ischemic stroke patients)	Acute ischemic stroke	LC-MS/MS	Post-stroke cognitive impairment (PSCI, MMSE)	Age, education level, hypertension, diabetes, NIHSS score, Hs-CRP	No
Liu D, 2023	China	Nested case-control	412 (Stroke cases) + 412 (Matched controls)	Total stroke, ischemic stroke, non-ischemic stroke	LC-MS/MS	Stroke risk (total, ischemic, non-ischemic)	BMI, smoking, hypertension, educational attainment, eGFR	Yes
Nie J, 2018	China	Nested case-control	622 (First stroke cases) + 622 (Matched controls)	First stroke (ischemic/hemorrhagic)	LC-MS/MS	First stroke risk	Choline, L-carnitine, baseline SBP, time-averaged SBP, eGFR	Yes
Sun T, 2020	China	Hospital-based case-control	953 (First ischemic stroke cases) + 953 (Matched controls)	First acute ischemic stroke	LC-MS/MS	Ischemic stroke risk	Age, sex, BMI, smoking, diabetes, hypertension, lipid indices (TG, LDL-C, HDL-C)	Yes
Guasch-Ferre M, 2017	Spain	Case-cohort (PREDIMED Trial)	229 (CVD cases, including stroke) + 751 (Sub-cohort)	Stroke (as part of composite CVD)	LC-MS/MS	CVD risk (including stroke), stroke risk	Age, sex, BMI, family history of CHD, smoking, physical activity, hypertension, diabetes	Yes
Rexidamu M, 2019	China	Case-control	255 (First-ever acute ischemic stroke) + 255 (Age/gender-matched healthy controls)	First-ever acute ischemic stroke	UHPLC-MS/MS	Stroke severity (NIHSS), infarct volume, Functional outcome (mRS at discharge)	Age, sex, BMI, stroke etiology (TOAST), vascular risk factors (Hypertension, diabetes, atrial fibrillation), infarct volume, Hs-CRP, FBG, HCY	No
Zhang J, 2021	China	Single-center prospective cohort	351 (First-ever acute ischemic stroke patients) + 150 (Age/gender-matched healthy controls)	First-ever acute ischemic stroke	LC-MS/MS	3-Month poor functional outcome (mRS 3–6), All-Cause mortality, stroke severity (NIHSS)	Age, sex, BMI, traditional vascular risk factors, NIHSS score, infarct volume, stroke subtype, pre-stroke/acute treatment, eGFR, glucose, CRP, IL-6, choline, betaine	No

LC-MS/MS, Liquid chromatography-tandem mass spectrometry; UHPLC-MS/MS, Ultrahigh performance liquid chromatography-tandem mass spectrometry; NIHSS, National Institutes of Health Stroke Scale; mRS, modified rankin scale; MMSE, mini-mental state examination; LAA, large artery atherosclerotic; eGFR, estimated glomerular filtration rate; SBP, systolic blood pressure; CVD, cardiovascular disease; TG, triglycerides; LDL-C, low-density lipoprotein cholesterol; HDL-C, high-density lipoprotein cholesterol; Hs-CRP, hypersensitive C-reactive protein; FBG, fasting blood glucose; HCY, homocysteine; IL-6, interleukin-6.

### 3.2 Risk of bias

The quality of the 11 included observational studies was evaluated using the NOS. The assessment showed that the NOS scores of all studies ranged from 6 to 8 out of a maximum of 9, indicating satisfactory overall methodological quality. Specifically, 9 studies (22–24, 26, 27, 31, 33–35) were rated as high quality (NOS score ≥ 7), while the remaining 2 studies (25, 28) had a score of 6, indicating moderate quality. Most studies scored highly in the “Selection” and “Outcome” (or “Exposure”) domains, reflecting rigorous definitions and ascertainment of cases and controls, as well as robust assessment methods. Points were primarily deducted in the “Comparability” domain, largely because some studies (25, 28, 33) did not adequately report on or control for key confounding variables (e.g., age, renal function). The detailed results of the quality assessment are presented in Supplementary Table 1.

### 3.3 Admission TMAO levels

**Figure 2:**
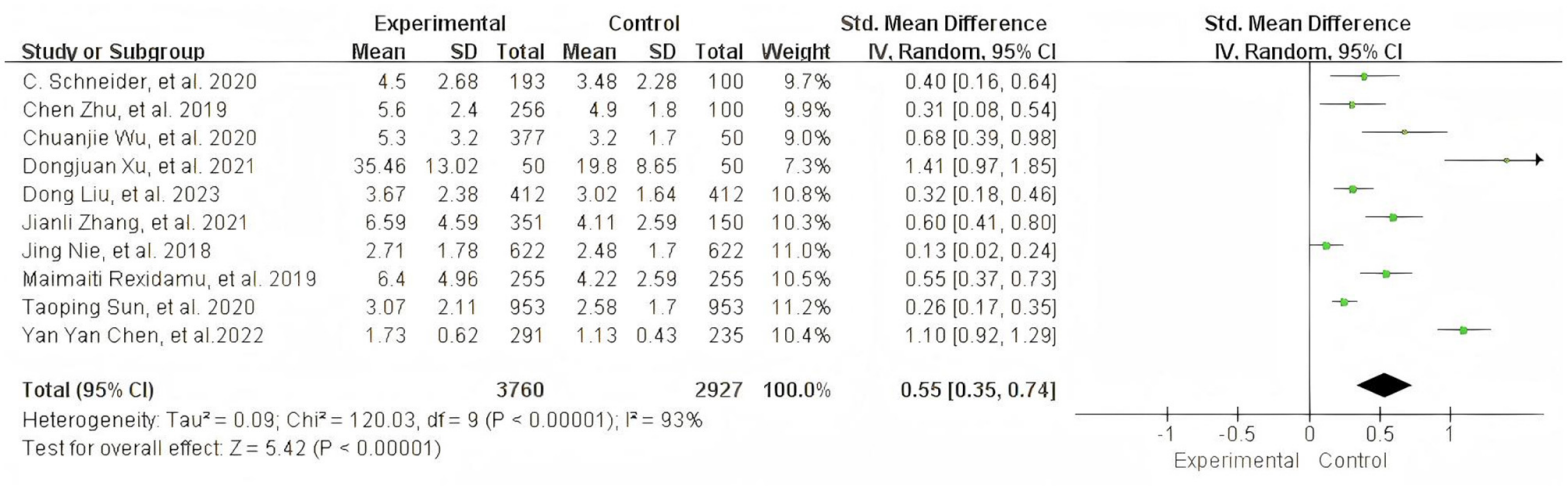
Meta-analysis of admission TMAO levels: forest plot and data summary.

10 studies (22–28, 33–35) reported blood TMAO levels at admission. Significant heterogeneity existed (*I*^2^ = 93%, likely stemming from different TMAO measurements and admission conditions), warranting a REM. Standardized Mean Difference (SMD) was used as the summary effect measure to better accommodate potential differences in measurement scales of raw data and uncertainties introduced by conversion [of these 10 studies, 6 (23–28) presented the admission TMAO levels by medians and quartiles, which were converted to the mean and SD by formulas of Wan et al. and Luo et al.]. Meta-analysis revealed significantly higher TMAO levels in stroke patients compared to controls (SMD = 0.55, 95% CI: 0.35, 0.74; *Z* = 5.42, *P* < 0.00001). Heterogeneity (*I*^2^ > 50%) likely stems from methodological variations in TMAO measurement and differences in the severity of patients' conditions at admission (Figure 2).

### 3.4 Dose-response meta-analysis

#### 3.4.1 Primary results

**Figure 3:**
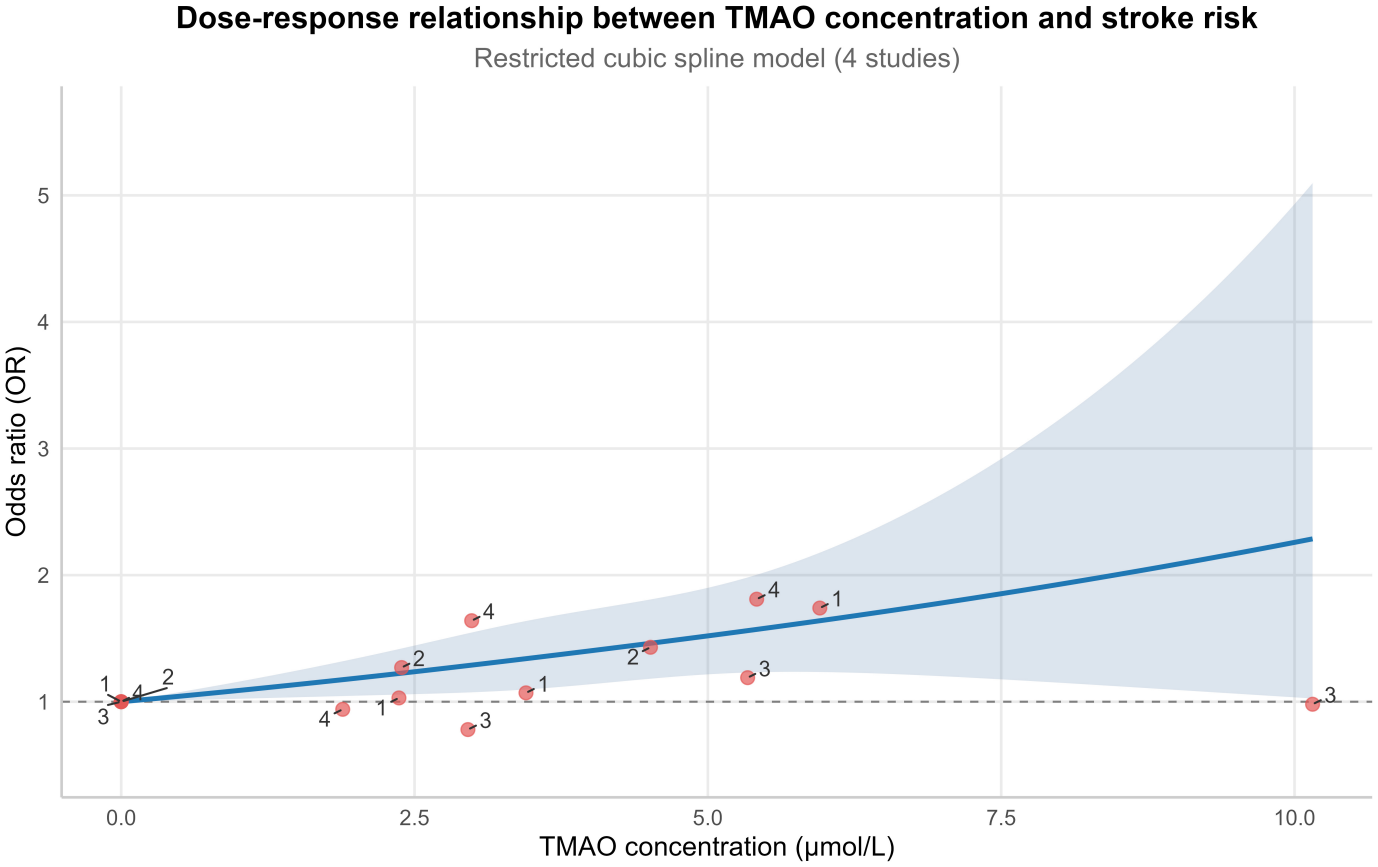
Model *p*-value = 0.0011 | *I*^2^ = 35.9%. Linear term: β = 0.0851 (95% CI: 0.0232, 0.1469), *p* = 0.0070. Non-linear term: β = −0.0065 (95% CI: −0.2012, 0.1883), *p* = 0.9479. Shaded area: 95% confidence Interval. Dose-response relationship between TMAO and stroke risk (RCS model).

**Figure 4:**
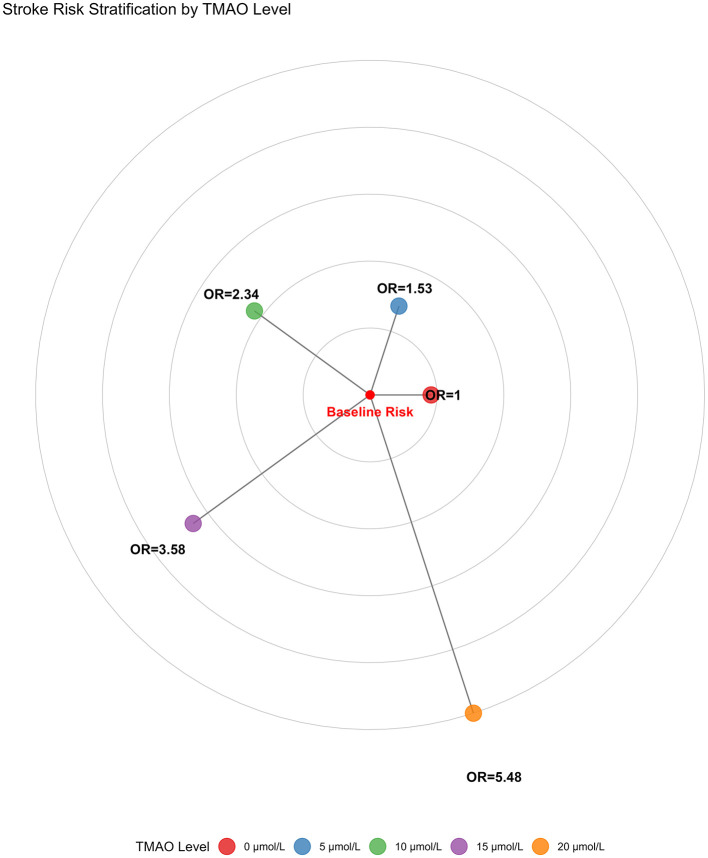
Cumulative increase in stroke risk with rising TMAO (radar chart).

4 studies (22, 23, 26, 31) were included in the DRMA. A significant dose-response relationship existed between TMAO and stroke risk (χ^2^ = 13.6170 (df = 2), *P* = 0.0011). Linear dose-effect: Each 1 μmol/L increase in TMAO was associated with a significant 0.0851 increase in the log-odds ratio (logOR) for stroke (95% CI: 0.0232, 0.1469; *P* = 0.0070), corresponding to an OR of 1.089 (95% CI: 1.023, 1.158). This indicates an 8.9% increased stroke risk per 1 μmol/L TMAO increment. Non-linearity was non-significant (*P* = 0.9479, 95% CI: −0.2012, 0.1883), supporting a linear trend. Moderate heterogeneity was observed (*I*^2^ = 35.9%; Cochran *Q*: *Q* = 9.3647, df = 6, *P* = 0.1541) (Detailed data are presented in Table 2). The RCS plot for the dose-response relationship is presented in Figure 3. Following the establishment of a significant dose-response relationship (model chi-square test *P* = 0.001), a key finding of our analysis was the identification of a TMAO risk threshold at 3.0 μmol/L. In addition, we calculated the cumulative risk increase and plot a radar chart (Figure 4) to visually demonstrate this risk accumulation.

**Table 2:** Results of the dose-response meta-analysis on TMAO and stroke risk.

**Parameter**	**Estimate**	**SE**	***z*-value**	***p*-value**	**95% CI**
**Fixed-effects coefficients**					
Linear term (β1)	0.0851	0.0315	2.70	0.007	(0.0232, 0.1469)
Nonlinear term (β_2_)	−0.0065	0.0994	−0.07	0.948	(−0.2012, 0.1883)
**Model fit and heterogeneity**	**Value**	**df**		***p*** **-value**	
Model Chi-square (χ^2^)	13.62	2		0.001	
Cochran's Q (Heterogeneity)	9.36	6		0.154	
*I*^2^ statistic	35.9%				
**Publication bias assessment**					
**Egger's test**		**SE**	***z*** **-value**	***p*** **-value**	**95%CI**
Intercept	0.097			0.070	(−0.015, 0.210)
**Trim-and-fill**					
Imputed studies (left side)	0	1.56			
Adjusted effect (β)	0.093	0.038	2.47	0.013	(0.019, 0.167)
Adjusted *I*^2^	46.7%			0.1314	

SE, standard error; CI, confidence interval; df, degrees of freedom.

#### 3.4.2 Sensitivity analysis

**Figure 5:**
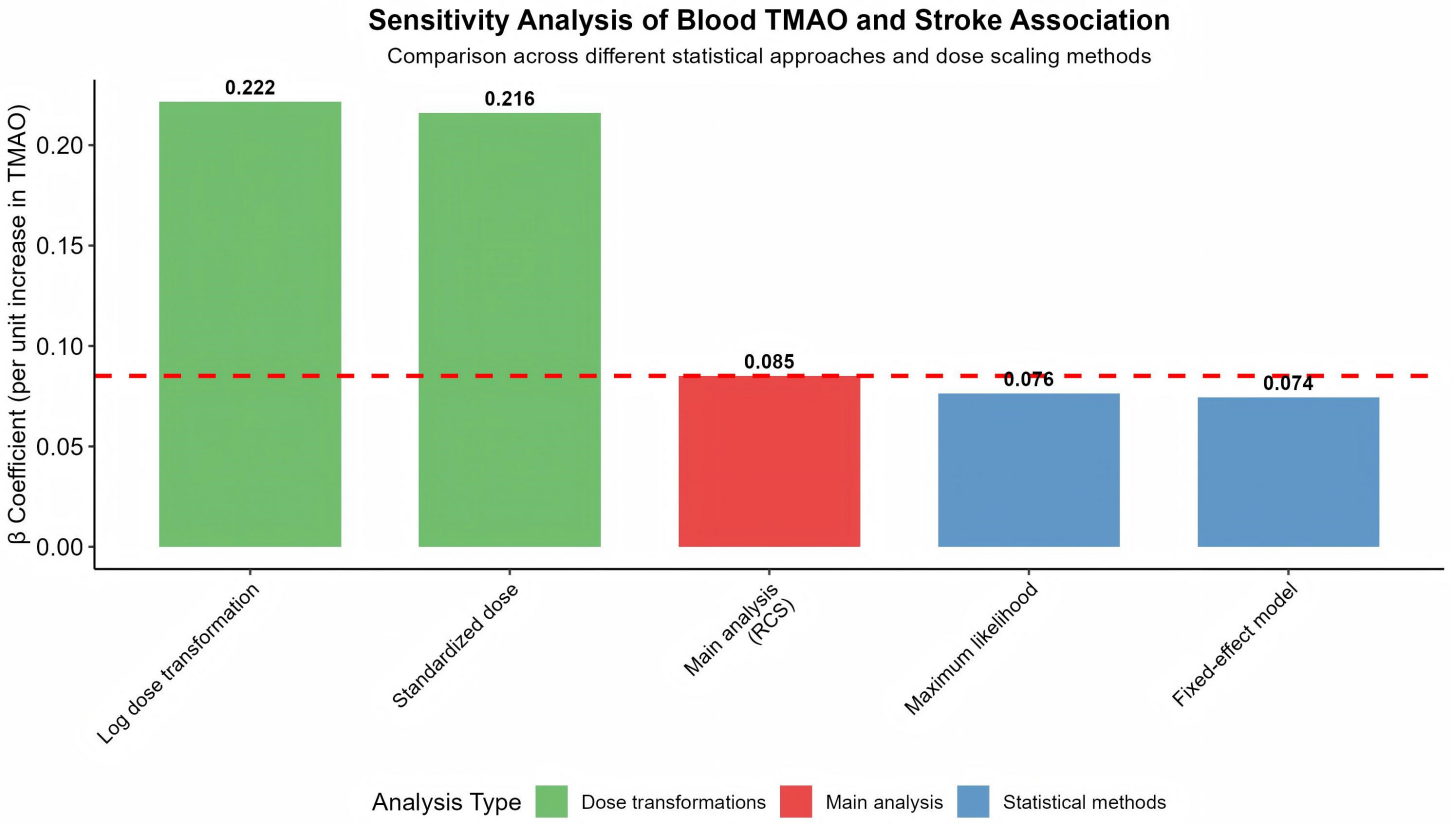
Sensitivity analysis of dose-response association. Red dashed line indicates the main dose-response meta analysis result (β = 0.0851).

Sensitivity analyses confirmed the robustness of the primary finding. As shown in Figure 5, all alternative approaches yielded a consistently positive association. The results from the FEM (β = 0.074) and maximum likelihood method (β = 0.076) were highly consistent with the primary analysis (β = 0.085). While point estimates from log-dose transformation (β = 0.222) and dose standardization (β = 0.216) were higher—reflecting the change in units of measurement—the direction of association remained unchanged. In summary, all pre-specified sensitivity analyses yielded results that corroborated the primary analysis, underscoring the robustness of the association between elevated blood TMAO levels and an increased risk of stroke.

#### 3.4.3 Publication bias

**Figure 6:**
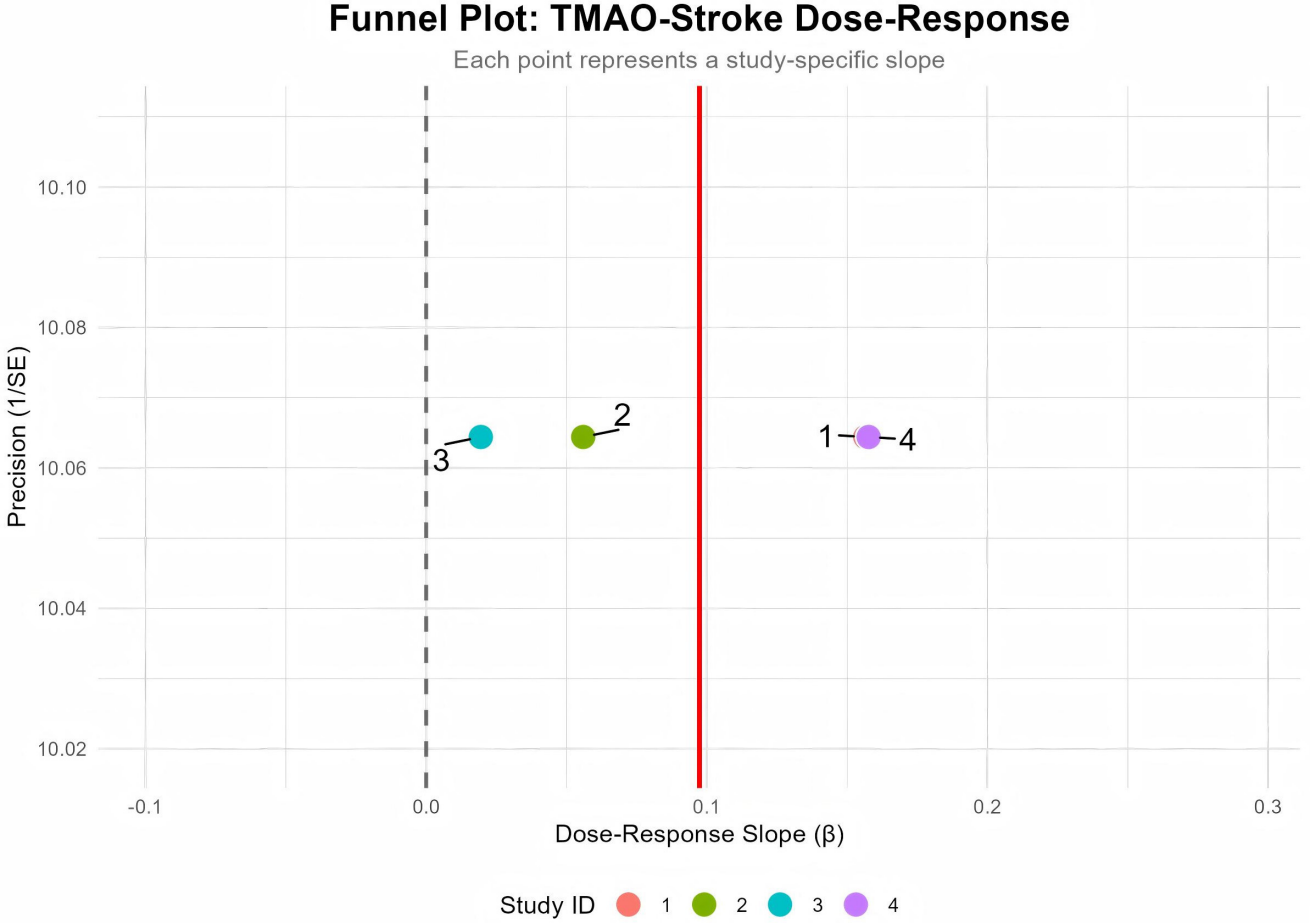
Funnel plot for publication bias assessment. Dashed line indicate 95% confidence interval boundaries.

**Figure 7:**
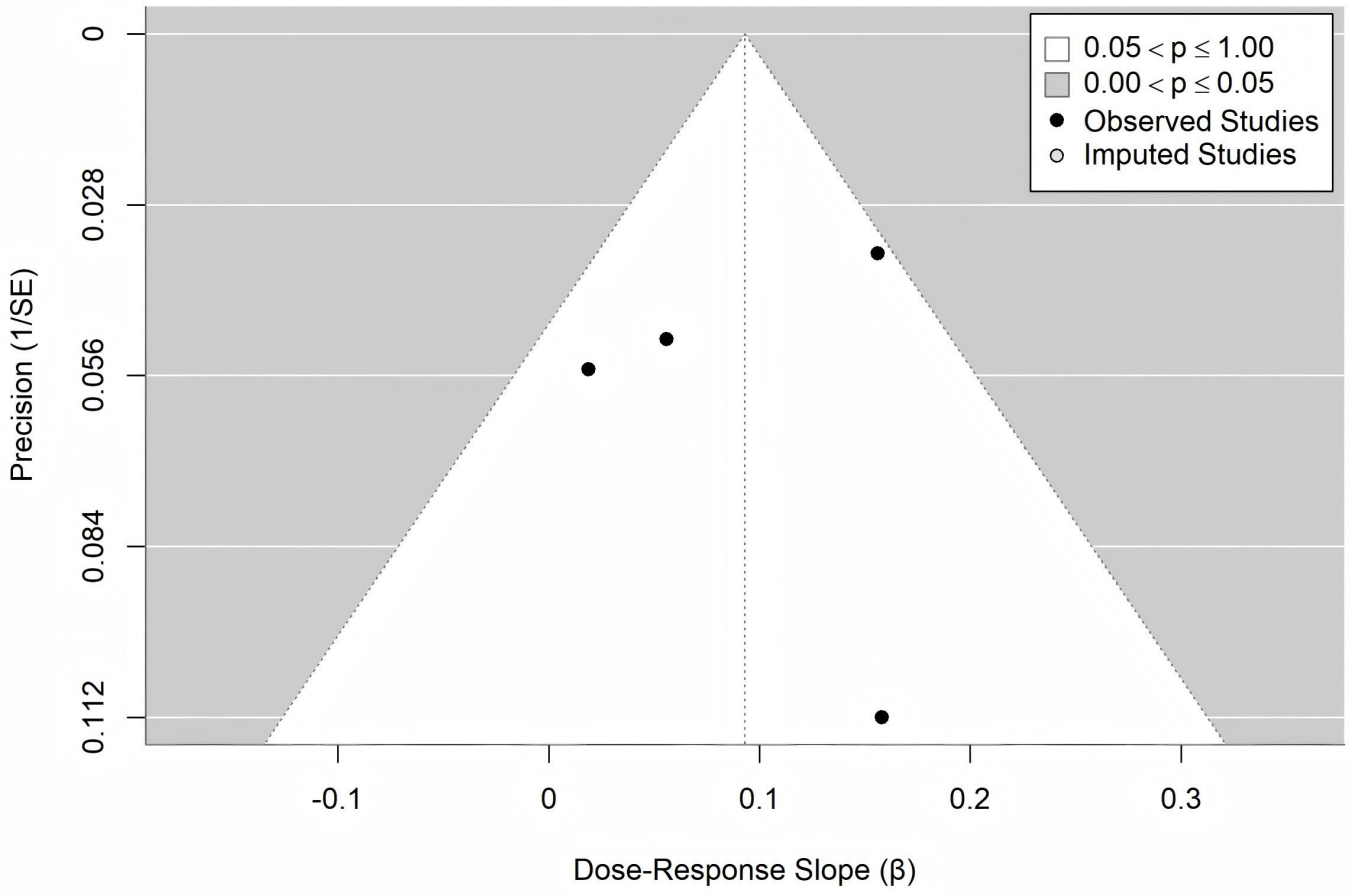
Trim-and-fill adjusted funnel plot.

Publication bias was assessed using a funnel plot (Figure 6) and Egger's test. Funnel plot visual inspection suggested symmetry. Egger's test showed borderline significance (Detailed data are presented in Table 2), potentially indicating limited power due to the small number of studies (*n* = 4). Further verification using the trim-and-fill (Figure 7) analysis indicated no missing studies (0 imputed, SE = 1.5649), yielding a consistent corrected slope β = 0.0931 (95% CI: 0.019, 0.167, *P* = 0.0134) (Detailed data are presented in Table 2).

## 4 Discussion

This study, utilizing a DRMA, has for the first time established and quantified a significant linear dose-response relationship between plasma TMAO levels and the risk of stroke. More importantly, we identified a critical clinical risk threshold, beyond which the stroke risk begins to demonstrate a statistically significant increase.

This finding holds substantial clinical translational value: it provides, for the first time, a quantifiable TMAO-based intervention threshold for stroke prevention. This enables clinicians to utilize TMAO as an objective biomarker for risk stratification in high-risk populations, facilitates the development of personalized prevention strategies, and allows for the dynamic assessment of the efficacy of interventions (such as dietary modifications or pharmacological treatments) during patient management. Wu et al. (36) reported a comparable threshold (4.29 μmol/L) in post-carotid stenting patients. While higher than our 3.0 μmol/L, both results confirm TMAO's promise as a risk marker for intervention. Additionally, multiple independent studies (17, 19) consistently report that each unit increase in TMAO significantly elevates stroke risk, consistent with our linear relationship. Among which, Chen and Guo (17), in their “Dose-Response Analysis” section, did test the possibility of a non-linear association. However, the statistical test result (*P* for non-linearity = 0.0738) was not significant. But a study (18) identified a non-linear relationship between TMAO and stroke risk, which may be attributed to the high heterogeneity of included studies (*I*^2^ = 99.7%, encompassing diverse populations, stroke types, and geographical regions) and the wide range of TMAO concentrations (0–20 μmol/L). In contrast, this study is the first to validate a specific linear risk threshold through a rigorous DRMA, which provides a clear intervention target for clinical practice, enabling physicians to stratify stroke risk and make preventive decisions based on TMAO levels, while other studies only reported risk increments or non-linear trends, lacking directly applicable thresholds. Secondly, the use of a two-stage random-effects model, Greenland–Longnecker covariance correction, and restricted cubic spline analysis ensured the robustness of the dose-response relationship. This robustness was further supported by sensitivity analyses. An exploratory assessment for publication bias was conducted (e.g., Egger's test), the interpretation of which is limited by the small number of studies. Notably, the pathophysiological role of TMAO extends beyond this. Hou et al. (37) found that elevated TMAO at admission independently predicts early neurological deterioration after stroke. Combined evidence positions TMAO as a persistent biomarker throughout stroke: lower pre-stroke thresholds (e.g., 3.0 μmol/L) may be applicable for primary prevention and risk stratification, while elevated levels in the acute phase may predict a worse clinical course.

In sensitivity analyses employing different statistical methods and dose-scaling strategies, the positive association between TMAO and stroke risk remained consistent, indicating that the primary finding is robust and not overly dependent on specific analytical assumptions. An apparent inconsistency was observed in the study by Guasch-Ferré et al. (31), which reported a decrease from an OR of 1.19 in Q3 to 0.98 in Q4. However, the wide and overlapping confidence intervals (Q3: 0.65–2.17; Q4: 0.53–1.84) suggest that this difference is not statistically significant and likely reflects random variation. Furthermore, the unique characteristics of the Mediterranean study population may also have contributed to this result. Their dietary patterns (rich in olive oil, nuts, and fish) may provide direct TMAO precursors (non-microbiota-dependent sources of TMAO) (38, 39). As noted by Caradonna et al. (8), diet custom can alter TMAO metabolism, explaining the heterogeneity in Mediterranean-diet population. Moreover, Hu et al. (40) also reported a non-significant association between TMAO levels and stroke incidence in the European subgroup. Publication bias was assessed. The funnel plot exhibited symmetry, and Egger's test was not significant, suggesting a low risk of bias. Moreover, the trim-and-fill correction did not impute any missing studies, and the effect size remained consistent after this procedure, further supporting the robustness of the results. Moderate heterogeneity, possibly due to population or methodological variations, did not affect the core conclusion. The positive dose-response relationship between TMAO and stroke risk is robust.

The strengths of this study include the establishment of a quantified linear dose-response relationship between TMAO and stroke risk, with 3.0 μmol/L identified as a clinical risk threshold. This finding was supported by robust statistical modeling utilizing a two-stage random-effects model with Greenland–Longnecker correction and restricted cubic spline testing. The analysis further demonstrated a clear risk stratification, revealing an accelerating risk gradient. Methodological rigor was ensured by excluding studies with sample sizes under 50 or incomplete adjustment for confounders, and potential publication bias was minimized through the application of trim-and-fill analysis.

However, this study is subject to several limitations. First, there is a notable regional bias in the included studies, with 9 out of 11 studies originating from China. Factors such as specific dietary patterns (e.g., Asian vs. Mediterranean diets) and gut microbiota composition may restrict the global generalizability of our findings. Second, there was insufficient control for potential confounders; for instance, acute-phase TMAO levels could be influenced by renal function or medication use, yet the included studies lacked standardized adjustment for these variables. Third, the dose-response analysis included only four studies, resulting in limited statistical power. Fourth, heterogeneity also stems from methodological differences in TMAO measurement, including the use of plasma vs. serum samples and varying timing of blood collection in the acute stroke phase. Finally, the literature search was restricted to Chinese and English databases, potentially introducing language bias.

Furthermore, this study could not explore diet-specific risk factors. Critically, the original studies included in our meta-analysis did not provide data on meat intake or other dietary components. Therefore, we could not directly establish the association between meat consumption and stroke risk within this analysis. Nonetheless, given that TMAO is primarily derived from dietary phosphatidylcholine and L-carnitine (abundant in red meat and processed meat), our results indirectly support the potential strategy of changing diet (e.g., reducing meat intake) to lower TMAO levels and thereby prevent stroke.

## 5 Conclusion

In summary, our analysis indicates a linear dose-response relationship between TMAO and stroke risk, identifying 3.0 μmol/L as a potential intervention threshold. Given the limitations such as regional bias and methodological variations, this finding requires further validation. Future research should prioritize standardized, large-scale multi-center studies to confirm these results and assess the clinical utility of TMAO monitoring in stroke prevention.
