# Supplementary material for: Dose-response meta-analysis of plasma TMAO and stroke: validated linear risk threshold at 3.0 μmol/L
Source: Front Neurol. 2026 Jan 30;17:1749522. doi: 10.3389/fneur.2026.1749522 (PMC12900722; doi:10.3389/fneur.2026.1749522)
Supplement: Supplementary file 1 [file Table_1.docx]

Supplementary Table 1. Newcastle - Ottawa Quality Assessment Scale

| First Author, Year | Study Type | Total NOS Score | Non-Compliant Items (×) |
| --- | --- | --- | --- |
| Liu D, 2023 | Case-Control Study | 8 | 1. Non-response rate: Different rates with no designation |
| Nie J, 2018 | Case-Control Study | 8 | 1. Non-response rate: Different rates with no designation |
| Sun T, 2020 | Case-Control Study | 8 | 1. Non-response rate: Different rates with no designation |
| Guasch-Ferre M, 2017 | Cohort Study | 8 | 1. Follow-up adequacy: Rate < adequate % with no description of lost subjects |
| Rexidamu M, 2019 | Case-Control Study | 7 | 1. Control selection: Hospital controls (not community controls)2. Non-response rate: Different rates with no designation |
| Zhang J, 2021 | Cohort Study | 6 | 1. Non-exposed cohort selection: From different source than exposed cohort2. Outcome assessment: Self-report (not independent blind assessment)3. Follow-up adequacy: No statement on follow-up status |
| Wu C, 2020 | Case-Control Study | 8 | 1. Non-response rate: Different rates with no designation |
| Schneider C, 2020 | Case-Control Study | 6 | 1. Control selection: Hospital controls (not community controls)2. Comparability: No control for additional factor3. Non-response rate: Different rates with no designation |
| Chen Y-Y, 2022 | Case-Control Study | 7 | 1. Control selection: Hospital controls (not community controls)2. Non-response rate: Different rates with no designation |
| Xu D, 2021 | Case-Control Study | 7 | 1. Control selection: No description of control source2. Non-response rate: Different rates with no designation |
| Zhu C, 2019 | Cohort Study | 8 | 1. Follow-up adequacy: Rate < adequate % with no description of lost subjects |
