# Supplementary material for: Dose-response meta-analysis of plasma TMAO and stroke: validated linear risk threshold at 3.0 μmol/L
Source: Front Neurol. 2026 Jan 30;17:1749522. doi: 10.3389/fneur.2026.1749522 (PMC12900722; doi:10.3389/fneur.2026.1749522)
Supplement: Supplementary file 2 [file Table_2.docx]

1. PubMed

#1 ("trimethyloxamine" [Supplementary Concept]) OR (((trimethyloxamine[Title/Abstract]) OR (TMAO[Title/Abstract])) OR (trimethylamine N-oxide[Title/Abstract]))

#2 ("Stroke"[Mesh]) OR ((((((((((((((Stroke[Title/Abstract]) OR (Strokes[Title/Abstract])) OR (Cerebrovascular Accident[Title/Abstract])) OR (Cerebrovascular Accidents[Title/Abstract])) OR (Cerebral Stroke[Title/Abstract])) OR (Cerebral Strokes[Title/Abstract])) OR (Cerebrovascular Apoplexy[Title/Abstract])) OR (Brain Vascular Accident[Title/Abstract])) OR (Brain Vascular Accidents[Title/Abstract])) OR (Cerebrovascular Stroke[Title/Abstract])) OR (Cerebrovascular Strokes[Title/Abstract])) OR (Apoplexy[Title/Abstract])) OR (CVA[Title/Abstract])) OR (CVAs[Title/Abstract]))

#3 ((((((((Observational Study[Title/Abstract]) OR (Cohort Study[Title/Abstract])) OR (Cohort Analysis[Title/Abstract])) OR (Cross Sectional Study[Title/Abstract])) OR (Cross Sectional Survey[Title/Abstract])) OR (Cross Sectional Analysis[Title/Abstract])) OR (Case-Control Study[Title/Abstract])) OR (Case-Comparison Study[Title/Abstract])) OR (Nested Case-Control Study[Title/Abstract])

#4 #1 and #2 and #3

1. Cochrane Library

#1 (trimethyloxamine):ti,ab,kw OR (trimethyloxamine):ti,ab,kw OR (TMAO):ti,ab,kw OR (trimethylamine N-oxide):ti,ab,kw

#2 (Stroke):ti,ab,kw OR (Strokes):ti,ab,kw OR (Cerebrovascular Accident):ti,ab,kw OR (Cerebrovascular Accidents):ti,ab,kw OR (Cerebral Stroke):ti,ab,kw OR (Cerebral Strokes):ti,ab,kw OR (Cerebrovascular Apoplexy):ti,ab,kw OR (Brain Vascular Accident):ti,ab,kw OR (Brain Vascular Accidents):ti,ab,kw OR (Cerebrovascular Stroke):ti,ab,kw OR (Cerebrovascular Strokes):ti,ab,kw OR (Apoplexy):ti,ab,kw OR (CVA):ti,ab,kw OR (CVAs):ti,ab,kw

#3 (Observational Study):ti,ab,kw OR (Cohort Study):ti,ab,kw OR (Cohort Analysis):ti,ab,kw OR (Cross Sectional Study):ti,ab,kw OR (Cross Sectional Survey):ti,ab,kw OR (Cross Sectional Analysis):ti,ab,kw OR (Case-Control Study):ti,ab,kw OR (Case-Comparison Study):ti,ab,kw OR (Nested Case-Control Study):ti,ab,kw

#4 #1 and #2 and #3

3. Web of Science

#1 ((TS=(trimethyloxamine)) OR TS=(TMAO)) OR TS=(trimethylamine N-oxide)

#2 (((((((((((((TS=(Stroke)) OR TS=(Strokes)) OR TS=(Cerebrovascular Accident)) OR TS=(Cerebrovascular Accidents)) OR TS=(Cerebral Stroke)) OR TS=(Cerebral Strokes)) OR TS=(Cerebrovascular Apoplexy)) OR TS=(Brain Vascular Accident)) OR TS=(Brain Vascular Accidents)) OR TS=(Cerebrovascular Stroke)) OR TS=(Cerebrovascular Strokes)) OR TS=(Apoplexy)) OR TS=(CVA)) OR TS=(CVAs)

#3 ((((((((TS=(Observational Study)) OR TS=(Cohort Study)) OR TS=(Cohort Analysis)) OR TS=(Cross Sectional Study)) OR TS=(Cross Sectional Survey)) OR TS=(Cross Sectional Analysis)) OR TS=(Case-Control Study)) OR TS=(Case-Comparison Study)) OR TS=(Nested Case-Control Study)

#4 #1 and #2 and #3

4. Embase

#1 'trimethyloxamine'/exp OR 'trimethyloxamine' OR 'tmao' OR 'trimethylamine n-oxide'/exp OR 'trimethylamine n-oxide'

#2 'stroke'/exp OR 'stroke' OR 'strokes' OR 'cerebrovascular accident'/exp OR 'cerebrovascular accident' OR 'cerebrovascular accidents' OR 'cerebral stroke' OR 'cerebral strokes' OR 'cerebrovascular apoplexy' OR 'brain vascular accident'/exp OR 'brain vascular accident' OR 'brain vascular accidents' OR 'cerebrovascular stroke' OR 'cerebrovascular strokes' OR 'apoplexy'/exp OR 'apoplexy' OR 'cva'/exp OR 'cva' OR 'cvas'

#3 'observational study'/exp OR 'observational study' OR 'cohort study'/exp OR 'cohort study' OR 'cohort analysis'/exp OR 'cohort analysis' OR 'cross sectional study'/exp OR 'cross sectional study' OR 'cross sectional survey' OR 'cross sectional analysis'/exp OR 'cross sectional analysis' OR 'case-control study'/exp OR 'case-control study' OR 'case-comparison study' OR 'nested case-control study'

#4 #1 and #2 and #3

5. Scopus

#1 TITLE-ABS-KEY ( trimethyloxamine ) OR TITLE-ABS-KEY ( TMAO ) OR TITLE-ABS-KEY ( trimethylamine AND N-oxide )

#2 TITLE-ABS-KEY ( Stroke ) OR TITLE-ABS-KEY ( Strokes ) OR TITLE-ABS-KEY ( Cerebrovascular AND Accident ) OR TITLE-ABS-KEY ( Cerebrovascular AND Accidents ) OR TITLE-ABS-KEY ( Cerebral AND Stroke ) OR TITLE-ABS-KEY ( Cerebral AND Strokes ) OR TITLE-ABS-KEY ( Cerebrovascular AND Apoplexy ) OR TITLE-ABS-KEY ( Brain AND Vascular AND Accident ) OR TITLE-ABS-KEY ( Brain AND Vascular AND Accidents ) OR TITLE-ABS-KEY ( Cerebrovascular AND Stroke ) OR TITLE-ABS-KEY ( Cerebrovascular AND Strokes ) OR TITLE-ABS-KEY ( Apoplexy ) OR TITLE-ABS-KEY ( CVA ) OR TITLE-ABS-KEY ( CVAs )

#3 TITLE-ABS-KEY ( Observational AND Study ) OR TITLE-ABS-KEY ( Cohort AND Study ) OR TITLE-ABS-KEY ( Cohort AND Analysis ) OR TITLE-ABS-KEY ( Cross AND Sectional AND Study ) OR TITLE-ABS-KEY ( Cross AND Sectional AND Survey ) OR TITLE-ABS-KEY ( Cross AND Sectional AND Analysis ) OR TITLE-ABS-KEY ( Case-Control AND Study ) OR TITLE-ABS-KEY ( Case-Comparison AND Study ) OR TITLE-ABS-KEY ( Nested AND Case-Control AND Study )

#4 #1 and #2 and #3

( TITLE-ABS-KEY ( trimethyloxamine ) OR TITLE-ABS-KEY ( TMAO ) OR TITLE-ABS-KEY ( trimethylamine AND N-oxide ) ) AND ( TITLE-ABS-KEY ( Stroke ) OR TITLE-ABS-KEY ( Strokes ) OR TITLE-ABS-KEY ( Cerebrovascular AND Accident ) OR TITLE-ABS-KEY ( Cerebrovascular AND Accidents ) OR TITLE-ABS-KEY ( Cerebral AND Stroke ) OR TITLE-ABS-KEY ( Cerebral AND Strokes ) OR TITLE-ABS-KEY ( Cerebrovascular AND Apoplexy ) OR TITLE-ABS-KEY ( Brain AND Vascular AND Accident ) OR TITLE-ABS-KEY ( Brain AND Vascular AND Accidents ) OR TITLE-ABS-KEY ( Cerebrovascular AND Stroke ) OR TITLE-ABS-KEY ( Cerebrovascular AND Strokes ) OR TITLE-ABS-KEY ( Apoplexy ) OR TITLE-ABS-KEY ( CVA ) OR TITLE-ABS-KEY ( CVAs ) ) AND ( TITLE-ABS-KEY ( Observational AND Study ) OR TITLE-ABS-KEY ( Cohort AND Study ) OR TITLE-ABS-KEY ( Cohort AND Analysis ) OR TITLE-ABS-KEY ( Cross AND Sectional AND Study ) OR TITLE-ABS-KEY ( Cross AND Sectional AND Survey ) OR TITLE-ABS-KEY ( Cross AND Sectional AND Analysis ) OR TITLE-ABS-KEY ( Case-Control AND Study ) OR TITLE-ABS-KEY ( Case-Comparison AND Study ) OR TITLE-ABS-KEY ( Nested AND Case-Control AND Study ) )

1. Sinomed

#1 "TMAO"[常用字段:智能] OR "氧化三甲胺"[常用字段:智能]

#2 "中风"[常用字段:智能] OR "脑卒中"[常用字段:智能]

#3 "观察性研究"[常用字段:智能] OR "队列研究"[常用字段:智能] OR "横断面研究"[常用字段:智能] OR "病例对照研究"[常用字段:智能]

#4 #1 and #2 and #3

1. CNKI

(TKA % 'TMAO' OR TKA % '氧化三甲胺' OR TKA % '三甲胺 N - 氧化物') AND (TKA % '卒中' OR TKA % '脑卒中' OR TKA % '脑血管意外' OR TKA % '中风' OR TKA % '脑中风') AND (TKA % '观察性研究' OR TKA % '队列研究' OR TKA % '横断面研究' OR TKA % '病例对照研究')

1. Wanfang

(主题:(氧化三甲胺 or TMAO or “三甲胺N - 氧化物”)) and (主题:(卒中 or 脑卒中 or 脑血管意外 or 中风 or 脑中风)) and (主题:(观察性研究 or 队列研究 or 横断面研究 or 病例对照研究))

1. VIP

(M=(氧化三甲胺 or TMAO or "三甲胺N - 氧化物")) and (M=(中风 or 脑中风 or 脑血管意外 or 卒中 or 脑卒中)) and (R=(观察性研究 or 队列研究 or 横断面研究 or 病例对照研究))
